# Radioresistance of human glioma spheroids and expression of HSP70, p53 and EGFr

**DOI:** 10.1186/1748-717X-6-156

**Published:** 2011-11-11

**Authors:** Carlos A Fedrigo, Ivana Grivicich, Daniel P Schunemann, Ivan M Chemale, Daiane dos Santos, Thais Jacovas, Patryck S Boschetti, Geraldo P Jotz, Aroldo Braga Filho, Adriana B da Rocha

**Affiliations:** 1 Laboratório de Marcadores de Estresse Celular, Universidade Luterana do Brasil, Canoas, RS, Brasil; 2 Programa de Pós Graduação em Diagnóstico Genético e Molecular, Universidade Luterana do Brasil, Canoas, RS, Brasil; 3 Programa de Pós Graduação em Genética e Toxicologia Aplicada, Universidade Luterana do Brasil, Canoas, RS, Brasil; 4 Serviço de Neurocirurgia do Hospital Beneficência de Porto Alegre, RS, Brasil; 5 Departamento de Ciências Morfológicas da Universidade Federal do Rio Grande do Sul, Porto Alegre, RS, Brasil; 6 Serviço de Radioterapia do Hospital São Lucas da Pontifícia Universidade Católica do Rio Grande do Sul, Porto Alegre, RS, Brasil

**Keywords:** Glioblastoma, spheroids, radioresistance, Hsp70, p53

## Abstract

**Background:**

Radiation therapy is routinely prescribed for high-grade malignant gliomas. However, the efficacy of this therapeutic modality is often limited by the occurrence of radioresistance, reflected as a diminished susceptibility of the irradiated cells to undergo cell death. Thus, cells have evolved an elegant system in response to ionizing radiation induced DNA damage, where p53, Hsp70 and/or EGFr may play an important role in the process. In the present study, we investigated whether the content of p53, Hsp70 and EGFr are associated to glioblastoma (GBM) cell radioresistance.

**Methods:**

Spheroids from U-87MG and MO59J cell lines as well as spheroids derived from primary culture of tumor tissue of one GBM patient (UGBM1) were irradiated (5, 10 and 20 Gy), their relative radioresistance were established and the p53, Hsp70 and EGFr contents were immunohistochemically determined. Moreover, we investigated whether EGFr-phospho-Akt and EGFr-MEK-ERK pathways can induce GBM radioresistance using inhibitors of activation of ERK (PD098059) and Akt (wortmannin).

**Results:**

At 5 Gy irradiation UGBM1 and U-87MG spheroids showed growth inhibition whereas the MO59J spheroid was relatively radioresistant. Overall, no significant changes in p53 and Hsp70 expression were found following 5 Gy irradiation treatment in all spheroids studied. The only difference observed in Hsp70 content was the periphery distribution in MO59J spheroids. However, 5 Gy treatment induced a significant increase on the EGFr levels in MO59J spheroids. Furthermore, treatment with inhibitors of activation of ERK (PD098059) and Akt (wortmannin) leads to radiosensitization of MO59J spheroids.

**Conclusions:**

These results indicate that the PI3K-Akt and MEK-ERK pathways triggered by EGFr confer GBM radioresistance.

## Background

Glioblastoma multiforme (GBM) is among the most radioresistant tumors [1]. The standard therapy for GBMs consists of surgery, fractionated radiotherapy with concomitant temozolamide (TMZ) followed by adjuvant TMZ. Although this approach showed a significant increase in median overall survival from 12.1 months for patients treated with radiotherapy alone to 14.6 months after the combination of radiotherapy and TMZ [2,3]. The modest increase in survival time after radiotherapy treatment has been ascribed to the high intrinsic resistance of the GBMs to ionizing radiation [1,4].

Several different culture models have been used to determine the intrinsic radiosensitivity of gliomas. These include monolayer cultures of glioma lines, both early and late passage after initial isolation and spheroids derived from these cell lines [5,6]. It is assumed that spheroid cultures can better predict the *in vivo *response compared to monolayer cultures, since cell-cell contact, variation in cell cycle, altered metabolism, and diffusion of nutrients and oxygen or drugs may influence the outcome [7,8].

When irradiated, many cancer cells undergo cell death by multiple mechanisms of cell death. The main form of cell death is mitotic catastrophe, which subsequent leads to cell death when cells are unable to go trough mitosis. Cells might survive the treatment, but lose their clonogenic capacity, leading to a reduction in clonogenic cell survival. The actual manifestation of cell death can occur as necrosis, apoptosis or authophagy [9]. Thus, cells have evolved an elegant system in response to ionizing radiation induced DNA damage, where *p53 *has been shown to play an important role in the process. However, the *p53 *gene is the most commonly mutated tumor suppressor gene in malignant gliomas [10], pointing towards *p53 *status against radiotherapy response [11]. Also, the high expression of members of the Hsp70 family (heat shock protein of 70 KDa) in high grade gliomas indicates a possible role of these proteins in resistance to cancer therapy [12]. The identification of EGFr amplification and mutation in GBM has led to important advances in demonstrating that the EGFr (in combination with other genetic alterations) is likely to play an important role in the pathogenesis of this disease and some studies have correlated their overexpression with radioresistance [13]. Indeed resistance to apoptosis results from changes at the genomic, transcriptional and post-transcriptional levels of proteins, protein kinases and their transcriptional factor effectors. The PI3K/Akt and the Ras/Raf/MEK/ERK signaling cascades play critical roles in the regulation of gene expression and prevention of apoptosis. Components of these pathways are mutated or aberrantly expressed in human cancer, notably GBMs [14,15].

Therefore, in the present study the effect of ionizing irradiation on the expression of p53, Hsp70 and EGFr was evaluated in GBM spheroids. To this end, spheroids from U-87MG and MO59J cell lines as well as spheroids derived from primary culture of tumor tissue of one GBM patient (UGBM1) were irradiated, their relative radioresistance established and the p53, Hsp70 and EGFr contents were immunohistochemically determined. Moreover, we investigated whether EGFr-phospho-Akt and EGFr-MEK-ERK pathways can promote GBM radioresistance.

## Methods

### Cell culture

The U-87MG and MO59J human GBM cell lines were obtained from the American Type Culture Collection (Rockville, MD, USA). The primary GBM cells, named GBM1 was obtained from a 49-years-old white man that suffers surgery and did not receive chemotherapy or radiotherapy prior to the surgery procedure. A tumor specimen was excised and used for tumor processing. The pathological diagnosis was GBM based on the histologic features of vascular proliferation, hypercellularity, mitotic figures, gemistocytic nuclei, and necrosis. The establishment of the primary cell culture was performed accordingly to Farr-Jones [16]. Briefly, after biopsy at least 3 mm of the pathological fragment was sent to the laboratory to be processed. Samples were then mechanical dissociate, dropping of the visible stroma and veins. The cells were suspend in trypsin-EDTA for 20 min, centrifuged for 1,400 rpm for 10 min and resuspended in 25 cm^2 ^flasks with DMEM/F12 supplemented with 20% fetal calf serum (FCS) and 4 times the prescribed concentration of non essential amino acids. During the primary culture we progressively reduced the FCS concentration to 10%, thus cells were maintained in complete medium consisting of DMEM containing 2% (w/v) L-glutamine and 10% (v/v) FCS, at a temperature of 37^o^C, a minimum relative humidity of 95%, and an atmosphere of 5% CO_2 _in air. For experiments, exponentially growing cells between passages 10 to 15 were detached from the culture flasks either using trypsin-EDTA, or by scraping with a rubber policeman. Cell viability greater than 95% was confirmed by trypan blue exclusion.

### Spheroid formation

Once the monolayer cultures became confluent the cells were trypsinized and spheroids were performed using the liquid overlay technique of Carlsson and Yuhas [17]. In brief, exponentially growing monolayer cells were trypsinized and 2x10^6 ^cells were seeded in Petri dishes pre-coated with 2% agarose solution mixed in 1:3 ratio with DMEM supplemented with 10% FCS. After 2 days round spheroids were formed and those with 200 μm diameter were collected, transferred and culture individually in agarose-coated wells of 24-well plates (agar 1.5%) with complete culture medium.

### Spheroid treatments and volume determination

The spheroids were irradiated with single doses (5, 10 and 20 Gy) (dose rate 1.14 Gy per minute) using a Telecobalt Theretron Phoenix SR 7510 linear accelerator (Philips, Eindhoven, The Netherlands), at a source-to-target distance of 70 cm. Irradiation was applied just after the harvesting and isolation of spheroids in 24-well plates. After treatment, the dishes were incubated at 37°C. For some experiments the 5 Gy radiotherapy was concomitant and followed by 48 hours treatment with gefitinib (50 μM) (Astra Zeneca, Macclesfield, Cheshire, UK), wortmannin (5 μM) (Calbiochem, San Diego, USA) or PD098059 (50 μM) (Calbiochem, San Diego, USA). The diameters of at least 12 spheroids were measured with an inverted microscope each day during 15 days and the spheroid volume was calculated in accordance to the formula V = 4/3 πr^3^, where r = ½ √ d1.d2 and d = diameter [18].

### Immunohistochemical

Spheroids with 200 μm or more were removed from culture plates, fixed and embedded in paraffin. For spheroid immunohistochemistry, paraffin-five-μm-thick sections were mounted on organosilane-coated slides and dried overnight at 37°C. Sections were deparaffinized in xylene, rehydrated in graded alcohol, and washed with distillated water. Then the sections were treated for antigen retrieval using citrate (10 mM, pH 6.0) for 20 min at boiling temperature, followed by 20-min cool-down in citrate buffer at room temperature. For monolayer immunohistochemistry, confluent cell culture slides were fixed on cold acetone for 10 min and dried at room temperature.

Immunohistochemical procedure was carried on accordingly to manufactures instructions (Vectastain ABC System; Dako, Vector, CA, USA). Briefly, endogenous peroxidase activity was quenched by incubation in 3% hydrogen peroxide-methanol solution. Thereafter, slides were incubated for 20 min in protein block serum-free (Dako, Carpinteria, CA, USA). The respective primary antibodies p53, Hsp70, EGFr and phospho-Akt (Dako, Carpinteria, CA, USA) were applied, and the slides incubated for 30 min at 37°C and overnight at 4°C in a humidity chamber. Subsequently, slides were incubated with biotinylated secondary antibody (Vector, CA, USA) for 30 min. After incubation with VECTASTAIN^® ^ABC Reagent for 30 min, peroxidase activity was developed with DAB Substrate-Chromogen System (Merck, WS, NJ, USA) identifying bound antibody. After a final wash in distilled water, the slides were lightly counterstained with hematoxylin, dehydrated in graded alcohol, cleared with xylene, and mounted with xylene-based permanent mounting medium.

For all specimens, control slides were processed identically and at the same time, except that primary antibody was not applied. Therefore, all differences between the experimental tissue and the control tissue are ultimately due to DAB identification of the relevant protein.

### Immunohistochemistry analysis

Images from three fields were captured from each section at × 400 magnification through a microscope-mounted digital camera (Sony Corp, Tokyo, Japan) built on a Leica/CME microscopic (Leica, Wetzlar, Germany). The images were saved TIFF format and transferred onto an image analysis computer workstation for further analysis.

The immunohistochemistry analyses were realized by direct visualization and the arbitrary scoring system was carried on accordingly to Schmidt *et al. *[19]. The score was made for both extent (percentage of positive tumor cells: 0%, score = 0; < 5%, score = 1; 5-20%, score = 2; 21-50%, score = 3; 51-75%, score = 4, > 75%, score 5) and intensity (absent, score = 0; weak, score = 1; moderate, score = 2; strong, score = 3). Both scores were multiplied to give a composite score (0-15) for each tumor cell culture.

### ERK phosphorylation assay

Phosphorylation of ERKs 1 and 2 were determined by probing immunoblots with an anti-phospho-ERK1/2 antibody [20,21]. These determinations provide information on the extent of phosphorylation and thus activation of total ERK by MEK. Thus, spheroids treated with rhEGF (25 ng/mL) (Gibco BRL-Life Technologies, Long Island, NY, USA), 5 Gy irradiation, gefitinib (50 μM) or irradiation plus gefitinib (50 μM) were lysed and centrifuged, and aliquots of the supernatants with equal protein contents were subjected to SDS-PAGE. Separated proteins were transferred to nitrocellulose filters, which were blocked overnight at 4°C with 20 mM Tris pH 7.7, 137 mM NaCl, and 0.05% (v/v) Tween 20 (TTBS) containing 5% (v/v) non-fat dry milk (MTTBS). The filters were rinsed with TTBS, and incubated for 4 h at room temperature with anti-phospho-ERK1/2 antibodies (New England Biolabs Inc, Beverly, MA, USA) diluted 1:2,000 in MTTBS. Following three rinses in TTBS, filters were incubated for 2 h at room temperature with peroxidase-conjugated anti-mouse IgG diluted 1:500 in MTTBS. Proteins were detected by enhanced chemiluminescence (using a horseradish peroxidase-catalyzed and luminol-based reaction). The blots were stripped for 5 min with 1 mM NaOH, thoroughly washed, blocked, and reprobed with 1:20,000 diluted anti-ERK1/2 antibodies (Sigma Chemical Company, St. Louis, MO, USA) that recognize total ERK1/2.

### Statistical Analyses

For the statistical analysis of spheroids, paired *t*-Student test was used. All experiments were carried out at least three times in triplicate. All analysis were performed with GraphPad Instat (version 3.05; GraphPad Software Inc.; San Diego, CA, USA).

## Results

### Effect of ionizing radiation on human GBM spheroids growth

**Figure 1:**
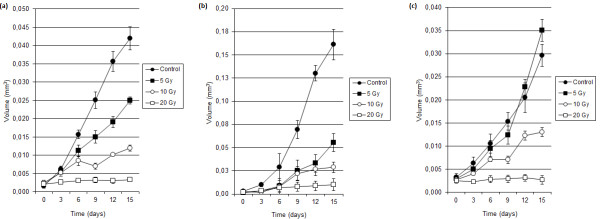
**Effects of irradiation on UGBM1 (a), U-87MG (b) and MO59J (c) human GBM spheroids proliferation**. Volume of non-irradiated or irradiated (5, 10 or 20 Gy) spheroids were determined every 3 days during a period of 15 days by measuring the spheroids diameter. Data was plotted as the mean ± SD of 24 GBM spheroids of three different experiments.

The volume growth of GBM spheroids after treatment with ionizing irradiation was determined. Escalating single doses of ionizing radiation (5, 10 and 20 Gy) promoted a dose-dependent decrease in growth for all three human GBM spheroids: UGBM1, U-87MG and M059J (Figure 1). The assays revealed a significant (p < 0.05) inhibition, within 72 h of 20 Gy irradiation of spheroid volume for all cell cultures. At lower doses irradiation (5 and 10 Gy) the UGBM1, U-87MG and M059J spheroids demonstrated different relative sensitivities. The spheroids that were relatively radiosensitive at these doses were U-87MG and UGBM1. These spheroids demonstrated a significant suppression of growth within 3 days of 5 Gy irradiation for U-87MG spheroids and within 9 days of 5 Gy irradiation for UGBM1 (p < 0.05). At day 15, the dose of 5 Gy irradiation reached 66% of reduction in U-87MG spheroid growth and 40% for UGBM1. Furthermore, 10 Gy irradiation significantly (p < 0.05) decreased the growth of U-87MG (within 24 h after irradiation, data not shown) and UGBM1 (within 6 days after irradiation) (Figure 1), inducing 82% of inhibition capacity in U-87MG and 71% in UGBM1 spheroids. While in MO59J spheroids the inhibition capacity (56%) was observed only at day 15. Thus, U-87MG spheroids were the most radiosensitive, while UGBM1 spheroids showed intermediate radiosensitivity. Conversely, MO59J spheroids presented relative radioresistance, when compared to U-87MG and UGBM1 spheroids. Inhibition of cell proliferation was observed only when MO59J spheroids were subjected to the higher irradiation doses (10 and 20 Gy) and after longer postradiation intervals (Figure 1).

### Effect of ionizing radiation on the p53 and Hsp70 contents on human GBM spheroids

Since the distinct sensitivity of the GBM spheroids studied to radiation-mediated toxicity might reflect differences in their susceptibility to undergo cell death, the effect of 5 Gy on p53 and the cytoprotective Hsp70 contents was first investigated.

**Figure 2:**
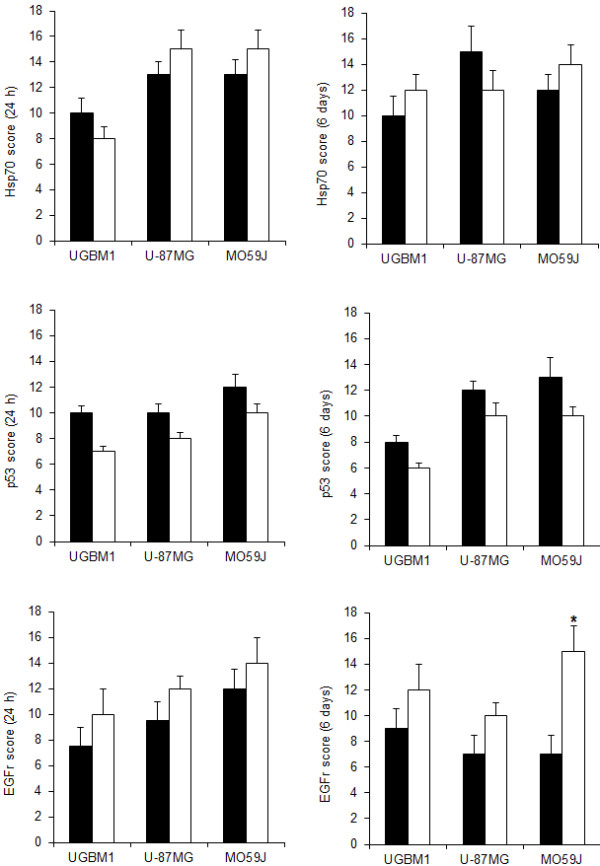
**p53, Hsp70 and EGFr immunohistochemical expression in UGBM1, U-87MG and MO59J spheroids following irradiation**. Immunostaining score was calculated for non-treated (control) or irradiated (5 Gy) as determined 24 h (left column) and 6 days (right column) after treatment. Arbitrary scoring system was calculated as described in Methods section. Data was plotted as the mean ± SD of three different experiments. *Significantly different from control (p < 0.05).

**Figure 3:**
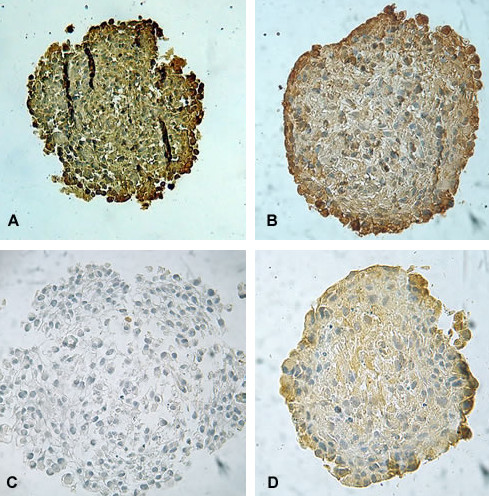
**Representative photomicrographs of the p53 content evaluated by immunohistochemistry in UGBM1 human GBM spheroids (A); Hsp 70 content following 5 Gy irradiation in MO59J spheroids (B); EGFr contents in no-irradiated (control) (C) and 5 Gy irradiated MO59J spheroids (D)**. EGFr antibody clone H11, recognizes wild-type EGFr and the deleted mutant form (EGFrvIII). Original magnification × 400.

In the spheroids established from UGBM1, U-87MG and MO59J cell cultures, the p53 expression was higher than 75% and the immunopositivity was found on the cytoplasm and nucleus. Also, p53 was uniformly distributed on all spheroids zone. No wide variety in the percentage of p53 immunoreactive tumor cells was documented between all three tumor cell spheroids. Overall, no significant changes in p53 expression were found following 5 Gy irradiation treatment in all spheroids studied (Figures 2 and 3).

At cellular level, immunohistochemistry assays reveal high accumulation of Hsp70 protein in all three GBM spheroids studied. Nevertheless, the location of the label varied with 5 Gy irradiation treatment, especially in MO59J spheroids. All control spheroids presented Hsp70 widely distributed on the spheroids. The irradiation did not induce significant alterations on the total Hsp70 contents on the GBM spheroids, but with 5 Gy treatment, MO59J spheroids showed a periphery distribution of the label on 6 days after irradiation (Figures 2 and 3). Taking into account that the high expression of Hsp70 on GBMs may induce resistance to cancer therapy [12,22] and that the ionizing radiation acts mainly in proliferating cells, which are mostly in the periphery of the tumor, it is possibly to suggest that Hsp70 can be involved on the MO59J GBM radioresistance.

### Effect of ionizing radiation on the EGFr signaling on human GBM spheroids

It is well known that the amplification of EGFr in GBM is related to the high cellular growth response. For this reason, we also investigated if the EGFr contents can be associated to the effect of 5 Gy irradiation on the spheroids. Then, immunohistochemistry analysis revealed high incidence of EGFr in all three GBM spheroids with more than 75% of incidence in the cytoplasm of the cells through all spheroid regions. Moreover, at 5 Gy irradiation treatment it was detected a significant increase on the EGFr levels in MO59J spheroids (p < 0.05) (Figures 2 and 3). This data suggest that EGFr is associated to GBM cellular response to radiotherapy, possibly protecting radioresistant cells against death.

**Figure 4:**
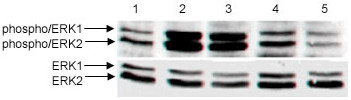
**Phosphorylation of ERK1/2 in MO59J spheroids after treatment with 5 Gy irradiation or gefitinib, alone or in combination**. ERK1/2 phosphorylation was determined by phospho-ERK immunoblotting using anti-phospho ERK1/2 antibody, and anti-ERK1/2 antibodies against total ERK1/2. Lane 1 - control; lane 2- rhEGF (25 ng/mL); lane 3 - 5 Gy irradiation, lane 4 - gefitinib (50 μM) and lane 5 - 5 Gy irradiation plus gefitinib (50 μM).

Radiation therapy may enhance the EGFr intracellular activation pathways, which in turn may induce proliferation, blockage of apoptosis and contribute to promote the tumor growth. Thus, we further examined whether the MEK-ERK cascade was involved in the transduction of the signals generated by EGFr in response to irradiation. To this end, ERK activation was assessed. In fact, 5 Gy irradiation promoted high phosphorylation on ERK1/2 in the same way as treatment with EGF in MO59J GBM spheroids. On opposite, Gefitinib treatment diminished the phosphorylation of the ERK1/2 (Figure 4), suggesting that EGFr-MEK-ERK signaling is involved on the GBM radiation response.

**Figure 5:**
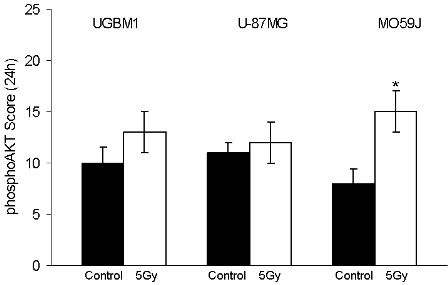
**Phospho-Akt immunohistochemical expression in UGBM1, U-87MG, and MO59J spheroids following irradiation**. Immunostaining score was calculated for non-treated control (■) or 5 Gy irradiated (□) as determined 24 h after treatment. Arbitrary scoring system was calculated as described in Methods section. Data was plotted as the mean ± SD of three different experiments. *Significantly different from control (p < 0.05).

Given that PI3K/Akt is another very important intracellular pathway involved in EGFr activation [23], Akt functions could trigger growth and antiapototic survival of GBM cells after irradiation. To test this hypothesis, we next examined whether phospho-Akt contents can be affected by ionizing radiation. A positive phospho-Akt immunostaining was detected in all spheroids samples. At 5 Gy irradiation the phospho-Akt content on MO59J spheroids presented an increase about 2 times (p < 0.05) (Figure 5). These data indicate that PI3K/Akt pathway is also associated to radiation response on the relative radioresistant MO59J spheroids.

**Figure 6:**
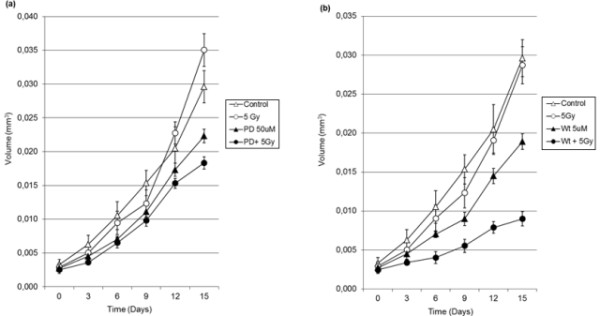
**MO59J human GBM spheroids proliferation after treatment with 5Gy irradiation, (a) PD098059 (50 μM), (b) wortmannin (5 μM), alone or in combination**. Volume of spheroids was determined every 3 days during a period of 15 days by measuring the spheroids diameter. Data was plotted as the mean ± SD of 24 GBM spheroids of three different experiments.

To evaluate mechanisms underlying the cellular response to ionizing radiation described above, we next examined the effect of the inhibition of the two main pathways of EGFr signaling, using a PI3K inhibitor - wortmannin and a MEK inhibitor- PD098059 on the MO59J spheroids growth after irradiation. Thus, 5 Gy irradiation treatment was concomitant and followed by 48 hours treatment with gefitinib (50 μM), wortmannin (5 μM) or PD098059 (50 μM). When PD098059 (50 μM) was added, the spheroids presented a significant decrease on their growth when compared to control. Combined radiation treatment showed significantly growth reduction of 40% (p < 0.05). Besides, the addition of the PI3K inhibitor - wortmannin (5 μM), which suppress the phosphorylation of Akt [24], significantly reduced the MO59J spheroids proliferation and a significant reduction of 68% the spheroid volume was observed in addition to 5 Gy irradiation (p < 0.05) (Figure 6). Together, these results suggest that the PI3K-Akt and MEK-ERK signaling are both triggering EGFr signaling against GBM radiotherapy effects.

## Discussion

It is widely accepted that the inherent radioresistance of some tumors is an important factor limiting for their curability. Clinical radioresistance of GBM has been demonstrated by local recurrence of the irradiated volume [25]. Then, an understanding of the molecular responses of GBM cells following irradiation may offer potential new targets for future therapy. In this study we have assessed the relative radiosensitivities of three types of GBM spheroids. We found that in response to radiation treatment, all cultures demonstrated a dose-dependent inhibition on cell proliferation. However, we could observe a clear difference in their radiosensitivity, which are in accordance with the clinical heterogeneity in GBM radiosensitivity.

It is assumed that spheroid cultures of glioma cell lines can better predict the *in vivo *response than monolayer cultures, since cell-cell contact, variation in cell cycle, altered metabolism, and diffusion of nutrients, oxygen or drugs may influence the outcome. The advantage of cell line spheroids is that they are relatively easy to obtain and to maintain in culture. Treatment-related changes of the growth kinetic of spheroids and the outgrowth of tumor cells present established and reproducible endpoints [26]. Using spheroids from biologically different human GBM cell lines and one from primary GBM culture, we have shown, as reported previously [27], that radiation consistently reduces the growth potential of all the GBM spheroids investigated. Another study examined whether, growth conditions also affect tumor cell radioresistance and radiation-induced DNA double strand breaks in a chromatin-dependent manner [28]. Essentially, that study showed that a 3D microenvironment results in increased tumor cell radioresistance mediated by less DNA double strand breaks and chromosomal aberrations since 3D culture conditions leads to increased amount of heterochromatin. In line with these findings, our data reveal that growth conditions contribute to the regulation of GBM cell fate and responsiveness to external stimuli since the U-87MG cell line was mentioned as a radioresistant in monolayer culture in a previous study from our lab and others [22]. While, the U-87MG spheroid model was more sensitive to ionizing radiation than MO59J spheroids. For instance, in MO59J spheroids, in which low radiation doses (5 and 10 Gy) are not significantly affecting cell proliferation, a permanent cell cycle or anti cell death mechanisms ensue.

GBM are generally lethal within 2 years of diagnosis due in part to the intense cell death resistance of its cancer cells, hence poor therapeutic response to radiotherapy. Alterations on the cell death pathways are generally believed to be at the basis of the resistance to ionizing radiation seen in many GBM patients [1]. The *p53 *tumor suppressor gene is frequently mutated in human malignances, including gliomas. In fact, the alterations of *p53 *gene play a significant role in the initiation and progression of astrocytomas [29-31]. Therefore, therapies aimed at restoring wild-type function or specifically targeting cells harbouring mutant *p53 *have been explored in preclinical models of gliomas [32-35], leading to clinical trial using adenovirus as gene delivery vector [36]. However, these strategies are controversial since some investigators have found that apoptosis can occur through alternative signaling pathways independent of *p53 *status [37-39]. In agreement, in our study, the irradiation treatment did not promote changes on p53 contents in all three GBM spheroids studied, which is also in accordance to most investigations that have found that *p53 *mutation or overexpression is not a significant prognostic factor for survival in GBM [40]. This may reflect the existence of other molecular alterations that abrogate *p53 *function, such as *mdm-2 *amplification or *p14^ARF ^*and *BCL2-like-12 *alterations [38,39,41]. Hence, it has been proposed that several events can be induced or suppressed to bring about cell death.

Solid tumors are generally stressed tissues, frequently expressing high levels of numerous proteins, especially members of the chaperone and heat shock protein (HSP) family. The most stress-inducible Hsp, Hsp70 (also known as Hsp72), is an antiapoptotic chaperone expressed abundantly in human tumors [42]. The tumorigenic potential of Hsp70 has been suggested to depend on its ability to transform cells and/or on its antiapoptotic properties [12]. In fact, in a previous study we have demonstrated that irradiation promotes increase of Hsp70 in radioresistant GBM cell line [22]. In the present study, the particularly high concentrations of Hsp70 on the periphery region of the spheroids following irradiation on the radioresistant MO59J spheroids could, at least in part, reflect a tolerance of the proliferating cells to survive despite of the stress conditions through activation of antiapoptotic pathways. In agreement to our data, it has been demonstrated that gene silencing by specific shRNA targeted against HSP70 resulted in significant inhibition of cell growth, G0/G1 arrest and increased apoptosis in the human colon cancer cell line HT29 [43].

Our findings suggest that EGFr may contribute to the radioresistance in GBMs. Several lines of evidence indicate that the EGFr signaling pathway may be an important factor in determining tumor cell response to ionizing radiation. EGFr blockage using a specific monoclonal antibody - C225, enhances radiosensitivity of U-87MG GBM cells [44]. Furthermore, the antitumor effect of anti-EGFr monoclonal antibody in combination with radiotherapy has been thought to result from the enhancement of the inhibition of EGFr signaling, increasing the cytotoxic effect of the radiation [13,45]. Consistent with these results we found that treatment with Gefitinib and irradiation diminished the ERK activation triggered by EGFr, supporting a notion that the EGFr signaling can be related to GBM radiation response [13].

The radiation therapy may enhance the EGFr intracellular activation pathways after treatment, which in turn may contribute against irradiation induced cell death. EGFr stimulation by the growth factor can lead to activation of phosphatidylinositol 3-kinase (PI3K), which catalyzes the conversion of phosphatidylinositol 4,5-biphosphate (PIP2) into phosphatidylinositol 3,4,5-triphosphate (PIP3). Finally, membrane-associated PIP3 attracts and activates the protein serine-threonine kinase Akt [46]. In fact, our results have shown an increase on the phospho Akt contents in the radioresistant MO59J spheroids. In addition, we found that the PI3K inhibitor - wortmannin leads to radiosensitization of these spheroids with a greater effect than the MEK inhibitor - PD098059. Thus, together, our data suggest that EGFr signaling induced by radiation is mediated by MEK-ERK pathway, but is mainly determined by PI3K-Akt signaling in the radioresistant GBM.

The identification of Akt as a key regulator of cellular survival has significant implications for current glioma biology [46]. Combined activation of Ras and Akt in neural progenitors induced GBM formation in mouse [47]. Elevated Ras activity and the phosphorylated Akt, as well as the deletion of PTEN, which downregulate Akt signaling, has been demonstrated in surgical specimens derived from human gliomas [48]. Therefore, deletion of active PTEN and overexpression of active Ras, combined with the overexpression of active PI3K, can renders cancer cells resistant to apoptosis by blocking adaptive cellular apoptosis through the hyperactivation of Akt [24].

In summary, the results of the current study demonstrate that EGFr signaling mediated by MEK-ERK and PI3K-Akt is involved in the response of GBM spheroids to radiation. Thus, we can propose that MEK-ERK and PI3K-Akt signaling are associated to protective effects against radiation induced cell death in radioresistant GBMs. Although the findings of this study cannot provide a mechanistic explanation to correlate these phenomena, we suggest that the protective role of EGFr signaling should be further investigated as a potential novel target to increase the sensitivity of human GBM to radiation.

## Conclusion

In conclusion, our findings indicate that the PI3K-Akt and MEK-ERK pathways may have a critical role in radiosensitivity of GBM cells. Therefore, selective inhibitors that specifically target PI3K-Akt and MEK-ERK signaling may have important therapeutic implications when used in combination with radiation in the treatment of GBM patients.

## Competing interests

The authors declare that they have no competing interests.

## Authors' contributions

CAF, DPS, DS, TJ and PSB performed experiments. IG, IMC, GPJ, ABF and ABR designed experiments, and IG and ABR wrote the manuscript. All authors have reviewed and approved the manuscript.
